# Supplementary material for: Strategies to reduce stigma and discrimination in sexual and reproductive healthcare settings: A mixed-methods systematic review
Source: PLOS Glob Public Health. 2022 Jun 15;2(6):e0000582. doi: 10.1371/journal.pgph.0000582 (PMC10021469; doi:10.1371/journal.pgph.0000582)
Supplement: S2 Table — (PDF) [file pgph.0000582.s002.pdf]

S4 Appendix. Risk of bias assessments for quantitative studies (ROBINS-I)

Author (year)	1. Bias due to confounding[1]	2. Bias in selection of participants[1]	3. Bias in classification of interventions[1]	4. Bias due to deviations from intended interventions[1]	5. Bias due to missing data[1]	6. Bias in measurement of outcomes[1]	7. Bias in selection of the reported result[1]	Overall risk of bias judgment[2]
Duby 2019	Critical<br>No control group or adjustment for potential confounders	Moderate<br>None	Moderate<br>None	No information<br>No information about deviations	No information<br>No information about missing data or loss-to-follow up	Serious<br>The outcome measure was subjective (i.e. vulnerable to influence by knowledge of the intervention received by study participants); and The outcome was assessed by assessors aware of the intervention received by study participants	No information<br>Unclear whether the reported results were selective	Critical
Geibel 2017	Critical<br>No control group or adjustment for potential confounders	Serious<br>Unclear how participants were recruited and why recruitment stopped after 400 participants	Moderate<br>None	No information<br>No information about deviations	Critical<br>25% loss to follow up from baseline to complete 3 surveys and 2 trainings	Serious<br>Participants knew they participated in the intervention	No information<br>Unclear whether the reported results were selective	Critical
Jadwin-Cakmak 2020	Critical<br>No control group or adjustment for potential confounders	Serious<br>Unclear how sites or participants were recruited	Moderate<br>None	No information<br>No information about deviations	No information<br>No information about missing data or loss-to-follow up	Serious<br>Participants knew they participated in the intervention	No information<br>Unclear whether the reported results were selective	Critical
Kinn 2003	Critical<br>No control group or adjustment for potential confounders	Serious<br>Only 17% and 8% of clients returned the questionnaires in 2000 and 2001 respectively	Moderate<br>None	No information<br>No information about deviations	Critical<br>49% of staff questionnaires returned blank. Unclear about missing data from client questionnaires.	Serious<br>Participants knew they participated in the intervention	No information<br>Unclear whether the reported results were selective	Critical
Maclean 2018	Critical<br>No control group or adjustment for potential confounders	Serious<br>Unclear how sites or participants were recruited	Moderate<br>None	No information<br>No information about deviations	No information<br>No information about missing data or loss-to-follow up	Serious<br>Participants knew they participated in the intervention	No information<br>Unclear whether the reported results were selective	Critical

Martin 2014	Critical<br>No control group or adjustment for potential confounders	Critical<br>Participants and sites self-selected into the intervention; no control group	Moderate<br>None	No information<br>No information about deviations	Serious<br>25% loss to follow up from baseline to second survey and 29% loss to follow up for endline	Serious<br>No control group so participants know they participated in the intervention	Low<br>None	Critical
Mosley 2020	Serious<br>No control group but adjustment for some potential confounders	Critical<br>Participants and sites self-selected into the intervention; no control group	Moderate<br>None	No information<br>No information about deviations	Serious<br>For the longitudinal analysis, loss to follow up of 12% and 19% in the two groups.	Serious<br>No control group so participants know they participated in the intervention	Low<br>None	Critical
Phiri 2019	Low<br>None	Low<br>None	Moderate<br>None	Serious<br>Stockouts during implementation at 10% of control and 20% of intervention facilities	Serious<br>Missing baseline data from at least 2 facilities; unclear % missing from social support and stigma numbers but missing data values assigned	Serious<br>Sites knew if they participated in the intervention	No information<br>Unclear whether the reported results were selective	Serious
Seybold 2014	Critical<br>No control group or adjustment for potential confounders	Critical<br>Participants self-selected to attend workshop	Moderate<br>None	No information<br>No information about deviations	Critical<br>74% of conference participants completed post-survey	Serious<br>Participants knew they participated in the intervention	No information<br>Unclear whether the reported results were selective	Critical

[1] No information, low, moderate, serious, critical

[2] Overall assessment: no information, low, moderate, serious, critical risk of bias
