# Supplementary material for: Strategies to reduce stigma and discrimination in sexual and reproductive healthcare settings: A mixed-methods systematic review
Source: PLOS Glob Public Health. 2022 Jun 15;2(6):e0000582. doi: 10.1371/journal.pgph.0000582 (PMC10021469; doi:10.1371/journal.pgph.0000582)
Supplement: S3 Table — (PDF) [file pgph.0000582.s003.pdf]

S5 Appendix. Critical appraisal of qualitative studies (CASP)

Author, year	Was there a statement of the aims of the research?[1]	Given the aim of the study, was a qualitative methodology appropriate?[1]	Was the research design appropriate to address the aims of the research?[1]	Were the data collected in a way that addressed the research issue?[1]	Was the recruitment strategy appropriate to the aims of the research?[1]	Was the relationship between the researcher and participants adequately considered?[1]	Have ethical issues been taken into consideration?[1]	Was the data analysis sufficiently rigorous?[1]	Were the findings supported by the evidence?[1]	Overall concerns[2]
**Harris 2011 & Debbink 2016**	Yes	Yes	Yes	Partial: Unclear where the workshops were facilitated, how they were conducted, & type of data generated. No discussion of saturation.	Yes	Yes	Yes	Partial: Limited discussion of the data analysis approach	Yes	Moderate concerns
**Littman 2009**	Yes	Yes	Yes	Yes	Yes	No: No discussion of reflexivity	Yes	Yes	Yes	Minor concerns

[1] Yes, no, partial or unclear. If no, partial or unclear, explain what is missing

[2] Overall assessment: no or very minor concerns, minor concerns, moderate concerns, serious concerns
