# Supplementary material for: Strategies to reduce stigma and discrimination in sexual and reproductive healthcare settings: A mixed-methods systematic review
Source: PLOS Glob Public Health. 2022 Jun 15;2(6):e0000582. doi: 10.1371/journal.pgph.0000582 (PMC10021469; doi:10.1371/journal.pgph.0000582)
Supplement: S1 Text — (PDF) [file pgph.0000582.s004.pdf]

S2 Appendix Review protocol

Interventions to reduce stigma and discrimination in sexual and reproductive healthcare settings: a systematic review and narrative synthesis: protocol

Background

Sexual and reproductive health and rights (SRHR) are integral components of health, social and economic development [1], but remain out of reach for many people globally. Political ideologies, social and cultural expectations around gender equality, reproductive choices and sexuality continue to threaten SRHR [1]. While SRHR are essential to achieve equitable, sustainable development [1], persistent inequities remain related to unintended pregnancy, pregnancy and childbirth complications, unsafe abortion, infertility, sexually transmitted infections (STIs), reproductive cancers, and gender-based violence. The 2030 Sustainable Development Agenda highlights two targets specific to SRHR: Target 3.7 to achieve universal access to sexual and reproductive healthcare services, and Target 5.6 ensuring universal access to SRHR [2]. While this ambitious agenda acknowledges universal access, more work is needed to ensure an equitable approach to SRHR to ensure that the needs and priorities of specific groups of people - who are persistently disadvantaged by existing systems of power - are not left behind.

The reproductive justice movement emphasizes this premise of seeking equity and ending oppression, and links SRHR to the social, political and economic inequalities that impact peoples' abilities to access these services [3]. Reproductive justice encourages the challenging of structural power inequality to engender change. This includes the cultural, social, and health care institutions and systems that produce and reproduce economic and health inequities [3]. Tantamount to achieving reproductive justice is acknowledging, then dismantling, oppressive systems that prohibit all people from achieving their fullest potential. Critical on this pathway is eliminating stigma and discrimination within healthcare settings.

Stigma and discrimination in healthcare settings

Stigma and discrimination are related concepts with distinct differences [4]. Link & Phelan (2001) define stigma as the co-occurrence of "labeling, stereotyping, separation, status loss and discrimination" in contexts where power is exercised [5]. Stigma is a fundamental cause of population health inequalities [4] for three key reasons [6, 7]: 1) stigma influences multiple disease outcomes through multiple risk factors; 2) stigma involves access to resources that can be used to avoid or minimize health risks or consequences; and 3) stigma is related to health inequalities irrespective of time or place. Central to the conceptualization of stigma is the context and experience of power, privilege and dominance that fosters environments of oppression and 'othering' of those who are stigmatized or discriminated against [8, 9].

Discrimination, on the other hand, has been defined as the unfair and unjust actions towards an individual or group on the basis of real or perceived status or attributes, a medical condition, socioeconomic status, gender, race, sexual identity, or age. Link and Phelan describe discrimination as the endpoint of the stigmatization process [5]. Others however view discrimination as manifestation of the stigmatization process [10]. Discrimination is said to be a fundamental feature and expression of stigma, occurring both at the structural-level (societal conditions constraining opportunity or well-being) and individual-level (unequal or unfair treatment based on membership of a social group) [4] however, stigma manifests in broader ways than discrimination alone. Stigma and discrimination manifest in broader society and in healthcare settings, as structures of healthcare

and reflect levels and types of privilege, power and disadvantage within society. When stigma or discrimination is experienced in healthcare settings, it is a violation of human rights [11, 12].

Stigma and discrimination depends on context, but there are common drivers, manifestations and consequences present across settings and populations [13]. Drivers of stigma in healthcare settings include health workers' fears, belief systems, negative attitudes, moral distress and lack of awareness about stigma, of a health condition or the population [13]. Institutional and broader health system and social policies may also drive stigma and discrimination in health settings [13]. Manifestations of stigma in healthcare settings are both overt and covert [13-15]. Research has consistently shown that individuals who experience sigma and discrimination in healthcare settings may delay or forego seeking health care in the future [16]. Once individuals from stigmatized groups get into the health system, they may be denied care or experience delays in receiving care. They may also be provided lower quality care, receive care that is not culturally appropriate, or experience mistreatment such as verbal and physical abuse [13, 17, 18]. All of these result in loss of trust in or satisfaction with the health system, resulting in a vicious cycle of not seeking care and delayed care seeking [19]. In addition, it leads to delayed diagnoses and initiation of care as well as lower adherence and engagement in care, leading to poor health outcomes [20, 21]. Moreover, the experience of discrimination itself has profound impacts on people's physical and mental health as well as their general wellbeing [22-24].

Structural and individual experiences or consequences of stigma and discrimination within sexual and reproductive healthcare have been well documented. Individuals who are disadvantaged by systems of power and seeking sexual and reproductive health services may receive unfair or unequal treatment, or have worse health outcomes compared to those who are in more privileged positions. In the United States, for instance, persistent disparities exist in maternal mortality where Black women are three times more likely to die than non-Hispanic white women, and these experiences occur within a history of racist reproductive policies [25]. For example, these reproductive rights policies intentionally practiced non-consensual or involuntary sterilization for Black women as a "prophylactic measure to…mitigate the menace of the 'unfit' and 'feebleminded'" [26]. Present-day disparities are driven by racism which manifests in various in various ways including Black women lacking access to sexual and reproductive services, disrespectful care and abuse in health care settings, and the general stress of living in race conscious societies [27-29].

Stigma and discrimination also drive inequities in sexual and reproductive outcomes based on women's social status, including socioeconomic status (SES), marital status, and age. Prior studies have shown women of low SES (measured variously by household wealth, caste, education, literacy, and employment status) are more likely to have poor experiences during childbirth than those of higher SES [30-32]. A WHO multi-country study in Ghana, Guinea, Myanmar and Nigeria showed that younger women were twice as likely to be physically abused and four times as likely to verbally abused during childbirth compared to older women [14]. Other forms of stigmatization based on gender and sexual orientation, disease conditions such as HIV, STIs, tuberculosis, leprosy, substance use, and mental illness as well as the type of care such as infertility and abortion services have been extensively documented as a barrier to care [17, 19, 30, 33-35].

Interventions to reduce stigma and discrimination in healthcare settings

Given the multi-level drivers and manifestations of stigma and discrimination, sustainable and scalable interventions to reduce stigma and discrimination in sexual and reproductive healthcare settings likely need to reflect this complexity. Nyblade and colleagues (2019) synthesized evidence

on facility-based interventions to reduce stigma in HIV, mental illness and substance abuse setting and identified six main strategies to reduce stigma broadly classified as [13]:

1. **Provision of information** to teach healthcare providers about health conditions, or stigma manifestations and consequences;
2. **Skills-building** for healthcare providers to improve competence in working with a stigmatized group;
3. **Participatory learning** for either/both healthcare providers and healthcare users to engage in the intervention;
4. **Contact with stigmatized groups** to humanize the stigmatized group and encourage healthcare providers to develop empathy;
5. **Empowerment approach** to improve healthcare user coping mechanisms to overcome stigma; and
6. **Structural or policy reform** to improve or create redress mechanisms, or facility restructuring.

Many interventions identified had multiple components to reduce stigma [13]. The mechanism of action for these interventions can be summarized as follows. Improving healthcare provider awareness of stigma and contact with the stigmatized group may translate into improved practices of empathy in clinical encounters. Training healthcare providers on concrete tools and approaches to address stigma and work with stigmatized groups transforms abstract theories of stigma into concrete action in how they provide care. Policy reform may create more enabling environments without structural disadvantages to certain groups seeking care. Lastly, using strengths-based and community-driven approaches can empower individuals and groups who are traditionally disadvantaged to achieve their SRHR by drawing on their own strengths and assets.

While experiences and impacts of stigma and discrimination in health have been increasingly well-documented, a critical gap remains in terms of interventions to reduce stigma and discrimination in sexual and reproductive health. We aim to address this gap by conducting a systematic review of interventions (inclusive of programs and policies) to reduce stigma and discrimination in sexual and reproductive healthcare. The overall aim of this review is to understand how interventions can be designed to reduce stigma and discrimination in sexual and reproductive healthcare services. The specific objectives are:

1. To describe interventions (type of intervention, design) to reduce stigma and discrimination in sexual and reproductive health services globally;

2. To assess the effectiveness of interventions to reduce stigma and discrimination in sexual and reproductive health services globally;

3. To analyze the characteristics of interventions shown to be effective and ineffective; and

4. To synthesize the key stakeholder (user, family member, community member, health worker, policy-maker) experiences and their perceptions of the feasibility, acceptability, and effectiveness of interventions to reduce stigma and discrimination in sexual and reproductive health services globally.

Mapping the research questions, types of data and analysis

	Research question	Type of data/analysis
**1.**	What types of interventions have been implemented to reduce discrimination in sexual and reproductive health services globally?	Narrative describing e.g. intervention/program components
**2.**	What is the effectiveness of interventions to reduce discrimination in sexual and reproductive health services globally?	Meta-analysis (if possible, but not likely), or narrative description e.g. of positive / negative impact.<br><br>Description of types of outcomes assessed in these interventions
**3.**	What are the characteristics of interventions shown to be effective and ineffective?	Logic model or some other appropriate presentation
**4.**	What are key stakeholder (user, family member, health worker) perspectives and experiences of interventions to reduce discrimination, in SRH services globally?	Qualitative or mixed-methods synthesis focusing on the user/provider side experiences of the interventions – likely from qualitative study, process evaluation, and/or surveys

Methods

Criteria for considering topics for this review

Types of studies

For objectives 1, 2, and 3, we will include randomized and non-randomized trials, pre-post studies (with or without a control group), interrupted time series, and other designs that compare the intervention to reduce discrimination with usual care. We will develop an operational definition of what constitutes an intervention that is inclusive of interventions using non-randomized designs and participatory approaches (e.g. workshops, sensitization training, simulation, mystery client). This review will focus on quantitative studies that have clear quantitative outcome measures. Intervention studies published in abstract form only are not eligible for inclusion, unless additional information can be obtained from the study authors. Where we identify study protocols, we will forward reference search to identify any results publications for relevance.

We will include studies that focus on interventions with the following characteristics:

1. Studies that directly aim to reduce stigma or discrimination in SRHR, or reducing inequity/promoting equity by reducing stigma and discrimination in SRHR;

2. Studies that include a quantitative outcome measure related to SRHR, regardless of type of outcomes (types of outcomes will be described in research question 2). We expect outcomes of interest to fall into five main categories related to both 1) reducing stigma, discrimination, or inequity in healthcare settings, and 2) improving outcomes in the causal pathway between the intervention and discrimination:
    a. Healthcare user or community experiences of stigma or discrimination,
    b. Healthcare user health and well-being outcomes,
    c. Healthcare access and utilization outcomes,
    d. Healthcare provider perceptions of stigma and discrimination, or
    e. Policy change or reform shown to reduce stigma, discrimination, or inequity.

3. Interventions that target the following population groups:
    a. Healthcare users or communities,
    b. Healthcare providers,
    c. Healthcare facilities,
    d. Health systems,
    e. Health laws or policies.

For objective 4, we will include primary studies that use qualitative, quantitative, or mixed-methods designs to evaluate user or provider experiences of interventions to reduce discrimination. This includes process evaluations, ethnographies, case studies, phenomenological studies, and surveys. We will include studies to meet objective 4 regardless of whether they were conducted alongside studies of effectiveness (e.g. sibling studies) included in objectives 1, 2, or 3. Qualitative studies do not need to have a comparison group to be included.

We will include both published and unpublished studies, and studies published in any language. We will not exclude studies based on our assessment of methodological limitations. We will use this information about methodological limitations to assess confidence in the review findings.

Topic of interest

We will include studies that focus on an intervention to reduce discrimination in sexual and reproductive healthcare settings. We have defined sexual and reproductive healthcare settings for this review as the following services, based on the Guttmacher–Lancet Commission on Sexual and Reproductive Health and Rights [1]:

- **Maternal health services**, including preconception care (pregnancy and infertility testing, counselling and services), antenatal care (including STI, gender-based violence screening, and preventing mother-to-child transmission (PMTCT)), childbirth care and postpartum care up to six weeks after birth for the baby and person giving birth (including immunization, breastfeeding)
- **Contraceptive counselling and services**, including STI screening
- **Safe abortion and post-abortion services**
- **Reproductive tract cancers including and cervical cancer counselling and services**

We have excluded STI and HIV counseling, testing, and treatment services if conducted outside the context of contraceptive or maternal health services, as these topics have been well documented in the literature [13, 20, 21]. We will use an intersectional approach to understanding discrimination by including studies that explore discrimination based on (but not limited to) race, ethnicity, Indigenous identity, social status, gender, sexuality, dis(ability), age, religion, migration/visa status, and the intersections between these identities.

Types of participants

We will include studies with all types of participants who are healthcare users, family members of healthcare users or community members, health workers of any cadre, policy-makers or other key stakeholders with no restrictions to sociodemographics or identity.

Settings

We will include studies conducted in any country globally. We will include studies where interventions target any type of setting where sexual and reproductive healthcare is received, which may include health facilities, community-based care, home-based care, or other types of institutional-based care.

Search methods for the identification of studies

We will search the following electronic databases: [MEDLINE, CINAHL, … - for discussion with UoM information specialist]. We will develop search strategies for each database, without any limits on language or publication date (Appendix 1). We will search all databases from inception to the date of search [to be added].

In addition to database searching, we will review the reference lists of all included studies and conduct a forward citation search for all included studies on Google Scholar. We will contact the authors of included studies to clarify published information and to seek unpublished data where necessary. We will contact researchers with expertise relevant to the review topic to request studies that might meet our inclusion criteria.

We will conduct a grey literature search in the following sources to identify studies not indexed in using OpenGrey (www.opengrey.eu.

Selection of studies

We will collate all title and abstracts identified from different searches into one reference database (EndNote) and remove duplicates. Two review authors will independently assess each record for its potential inclusion eligibility based on predefined criteria using Covidence. We will exclude references that do not meet the eligibility criteria. Then we will retrieve the full text of all the studies identified as potentially relevant after the title and abstract screening. All full texts will be assessed by two independent review authors for eligibility using Covidence. We will resolve any disagreements between two authors through discussion and consensus, or refer to a third reviewer for a final decision. If required, we will contact the study authors for further information to determine study eligibility. A PRISMA flow diagram illustrating our search results and the process of screening and selecting studies for inclusion will be developed.

Language translation

For title and abstract assessment of studies published in languages that none of the review team are fluent in (e.g. languages other than English, Spanish, French, Persian), we will carry out initial translation through open source software (Google Translate). If the translation indicates agreement with the inclusion criteria, or if the translation is insufficient to decide, we will ask other colleagues in our networks to assist in assessing full text for inclusion. If this cannot be done, we will categorize the study as 'studies awaiting classification' to ensure transparency in the review process.

Sampling of studies

To meet objectives 1-3, we will include an exhaustive list of studies meeting the eligibility criteria. To meet objective 4, we expect that most studies contributing relevant data will be qualitative studies. The synthesis to meet objective 4 will therefore likely be qualitative synthesis, and following the principles of qualitative evidence synthesis, will aim for both variation in concepts and depth of understanding of emergent themes, rather than an exhaustive sample, as the quality of the analysis can be threatened by large amounts of study data [36]. Once we have identified all studies eligible for to meet the objective 4, we will assess whether the number of studies or data richness is likely to represent a problem for the analysis and will consider selecting a sample of studies using a maximum variation purposive sampling approach based on study design, study aims, type of participants, country/region, and level of richness. If sampling is used, we will develop a sampling frame upon determining all variables and all eligible studies will be arranged within the frame. We will then review the studies in each frame to determine which studies we will include in the synthesis for objective 4. The sampling frame and any included but not sampled studies will be reported in a table.

Data extraction
We will use a form designed for this review to extract data on study setting, sample characteristics, objectives, guiding frameworks, study design, intervention design and components, data collection tools and analysis methods, and author conclusions. For intervention studies (objectives 1-3), we will extract data on the primary and secondary outcomes of interest related to discrimination (e.g. odds ratios, relative risks, prevalence estimates). We will operationally define these outcomes as part of the narrative synthesis process, as we expect substantial heterogeneity in outcome definition and assessment.

For qualitative studies assessing perceptions or experiences of the intervention (objective 4), we will extract the author themes, qualitative findings and participant quotations. For quantitative studies assessing perceptions or experiences of the intervention (objective 4), we will extract the primary and secondary outcomes of interest related to experiences or perceptions of discrimination.

Assessing the methodological limitations of included studies
Two independent review authors will critically appraise the quality of included studies using different tools depending on the study design:

- **Randomized interventional studies**: Cochrane RoB-2 tool
- **Non-randomized interventional studies**: Cochrane ROBINS-I tool
- **Observational studies:** Newcastle-Ottawa Scale
- **Qualitative studies:** adaptation of the Critical Appraisal Skills Programme (CASP) tool (www.casp-ul.net)

Any disagreement between review authors will be resolved through discussion or involving a third review author where necessary. We will report our assessments in a methodological limitations table.

Data management, analysis and synthesis
We will use a narrative synthesis approach [37], which is a particularly useful approach when analyzing data from different types of studies as it focuses on interpretive synthesis of the narrative findings of research, in order to sort and analyze studies into more homogenous groups based on critical components such as study design, study setting, program components, types of participants, type of health service and health topic, types of outcome, and direction or magnitude of effect of the intervention. There are four key elements of narrative synthesis recommended for implementation-focused narrative reviews, and these elements are analyzed iteratively throughout the review and synthesis process [37]:

1. ***Developing a preliminary synthesis***: we will develop an initial textual descriptive analysis of included intervention studies and their findings. This preliminary synthesis will allow us to identify and evaluate initial factors, components, and processes that may impact how we construct the analytic outputs from the subsequent analysis steps. We anticipate that meta-analysis will not be possible due to likely high heterogeneity of quantitative outcome data. Instead, we will use the textual descriptive analysis to group and cluster extracted data based on similar features, such as type of intervention, study location and context, community engagement, type of participants, and type of health service. We expect that key outputs from this process will include:
    a. Table 1: study summaries, and this will meet objective 1 (description of intervention/program components).

    b. Table 2: key effectiveness outcomes reported by study with meta-analysis (if possible), or logical groupings based on e.g. positive/negative/neutral direction of

effect, and description of types of outcomes assessed (e.g. self-report, indicator). This will meet objective 2 (effectiveness of interventions)

2. ***Exploring the relationships between studies***: narrative synthesis approaches are flexible and allow for iterative analysis based on emergent patterns and themes. In this phase we plan to explore the relationships between and across studies to understand how critical design factors may influence the likelihood of implementation success. Key relationships of interest include the relationships between study designs, levels of engagement, and magnitude/directionality of key findings. We expect this analysis to inform the development of a theoretical model (next phase) as well as implications for research and practice (Discussion section).

3. ***Mixed-methods synthesis of user experiences and perceptions***: In this phase, we plan to conduct the mixed-methods synthesis of key stakeholder perspectives and experiences of interventions to reduce discrimination. We will use a thematic analysis approach [38] and follow Cochrane EPOC guidance for conducting qualitative evidence syntheses [36]. This includes line-by-line coding of findings of primary studies, and organization into descriptive and analytic themes and interpretations [39]. We expect that key outputs from this process would include:
    a. Table 3: Summary of qualitative findings including assessments of confidence in the evidence, and explanations of these assessments, based on the GRADE-CERQual approach [40, 41]. This qualitative synthesis would meet objective 4.

    b. Matrix model: we will explore if a matrix model is a useful approach to depict whether the interventions included in the intervention review contain features or components valued by users or healthcare providers. If we use this approach, we will model based on previous complex reviews [42-44], to organize key features viewed as important moderators (positive or negative). This matrix model would contribute to objective 3.

4. ***Developing a theoretical model***: we plan to develop a logic model (objective 3) to link findings from the key stakeholder perceptions and experiences of interventions to reduce discrimination (objective 4) to the intervention/program components (objective 1), and the effect of the interventions (objective 2). The aim of this analytic element is to depict theories and assumptions about the links between elements of interventions, and health and well-being outcomes and experiences, rather than to demonstrate causal links between effectiveness and experience data. We will depict the logic model as a logical flow from components of the discrimination interventions, to intermediate or process outcomes, and resulting in longer-term outcomes, using similar methods described in Cochrane EPOC [36, 42, 45].

5. ***Assessing the robustness of the synthesis***: We will assess the robustness of the synthesis using multiple iterative methods to reflect on the methodological quality of the primary studies included in the synthesis and the trustworthiness of our analysis [37]. As described above, we will assess the methodological limitations of included studies using different tools appropriate for different study designs. We will consider throughout the analysis process how to minimize bias, for example by ensuring that studies of equal technical quality are given equal weighting, and by clearly stating eligibility criteria across each step of the review.

    We will assess the confidence in our qualitative review findings from step 3 using the GRADE-CERQual approach [40, 41] across the following domains: methodological limitations of included studies [46], coherence of the review findings [47], adequacy of data [48], and

relevance [49]. We will report these assessments transparently in a summary of qualitative findings table and evidence profiles.

6. ***Critical reflexivity of the review authors***: Throughout all stages of this review, we will practice critical reflexivity both as individuals and as a review team, which will also contribute to improving robustness of the synthesis. This enables us to consider, acknowledge and reflect on how our own lived experiences, employment, training, perspectives on discrimination and sexual and reproductive health services, and other factors shape and influence how we design and conduct the review, synthesis and interpretation of findings. We note at the start of this review that the strength of our team comes from our diversity: we have professional expertise and experience in sexual and reproductive health, public health, social sciences, medicine, obstetrics, epidemiology, social and reproductive justice, global health, and First Nations health. We currently work at academic institutions and organizations in high-resource settings, and our projects regularly engage with people who are disadvantaged by existing systems of power at both global and local levels. We may choose to present documentation of our reflexive practice in an appendix.

Acknowledgements

[to be added]

Sources of funding

This research is funded by the UNDP/UNFPA/UNICEF/WHO/World Bank Special Programme of Research, Development and Research Training in Human Reproduction (HRP), Department of Sexual and Reproductive Health and Research, World Health Organization. MAB's time is supported by an Australian Research Council Discovery Early Career Researcher Award (DE200100264) and a Dame Kate Campbell Fellowship. PA's time is partially supported by a Eunice Kennedy Shriver National Institute of Child Health & Human Development Career development award (R00HD093798). AW's time is supported by a National Health & Medical Research Council Postgraduate Scholarship.

References

1. Starrs AM, Ezeh AC, Barker G, Basu A, Bertrand JT, Blum R, et al. Accelerate progress - sexual and reproductive health and rights for all: report of the Guttmacher - Lancet Commission. The Lancet. 2018;391(10140):2642-92.
2. United Nations. Sustainable Development Goals 2017 [Available from: http://www.un.org/sustainabledevelopment/sustainable-development-goals/.
3. Ross LJ. Reproductive Justice as Intersectional Feminist Activism. Souls. 2017;19(3):286-314.
4. Hatzenbuehler ML, Phelan JC, Link BG. Stigma as a fundamental cause of population health inequalities. American journal of public health. 2013;103(5):813-21.
5. Link BG, Phelan JC. Conceptualizing Stigma. Annual Review of Sociology. 2001;27(1):363-85.
6. Phelan JC, Link BG, Tehranifar P. Social conditions as fundamental causes of health inequalities: theory, evidence, and policy implications. Journal of health and social behavior. 2010;51 Suppl:S28-40.
7. Link BG, Phelan J. Social conditions as fundamental causes of disease. Journal of health and social behavior. 1995;Spec No:80-94.
8. Hussein J, Ferguson L. Eliminating stigma and discrimination in sexual and reproductive health care: a public health imperative. Sexual and Reproductive Health Matters. 2019;27(3):1-5.
9. (UNAIDS) JUNPoHA. Protocol for the identification of discrimination against people living with HIV. Geneva, Switzerland: UNAIDS; 2000.
10. Nayar US, Stangl AL, De Zalduondo B, Brady LM. Reducing stigma and discrimination to improve child health and survival in low- and middle-income countries: promising approaches and implications for future research. Journal of health communication. 2014;19 Suppl 1(sup1):142-63.
11. Khosla R, Zampas C, Vogel JP, Bohren MA, Roseman M, Erdman JN. International Human Rights and the Mistreatment of Women During Childbirth. Health and Human Rights Journal. 2016;18(2):131-43.
12. Zampas C, Amin A, O'Hanlon L, Bjerregaard A, Mehrtash H, Khosla R, et al. Operationalizing a Human Rights-Based Approach to Address Mistreatment against Women during Childbirth. Health and Human Rights Journal. 2020;June 8, 2020.
13. Nyblade L, Stockton MA, Giger K, Bond V, Ekstrand ML, Lean RM, et al. Stigma in health facilities: why it matters and how we can change it. BMC Medicine. 2019;17(1):25.
14. Bohren MA, Mehrtash H, Fawole B, Maung TM, Balde MD, Maya E, et al. How women are treated during facility-based childbirth in four countries: a cross-sectional study with labour observations and community-based surveys. Lancet. 2019.
15. Bohren MA, Vogel JP, Hunter EC, Lutsiv O, Makh SK, Souza JP, et al. The Mistreatment of Women during Childbirth in Health Facilities Globally: A Mixed-Methods Systematic Review. PLoS Med. 2015;12(6).
16. Rüsch N, Angermeyer MC, Corrigan PW. [The stigma of mental illness: concepts, forms, and consequences]. Psychiatrische Praxis. 2005;32(5):221-32.
17. Ross CA, Goldner EM. Stigma, negative attitudes and discrimination towards mental illness within the nursing profession: a review of the literature. Journal of psychiatric and mental health nursing. 2009;16(6):558-67.
18. Dodor EA, Kelly S, Neal K. Health professionals as stigmatisers of tuberculosis: insights from community members and patients with TB in an urban district in Ghana. Psychol Health Med. 2009;14(3):301-10.
19. van Boekel LC, Brouwers EP, van Weeghel J, Garretsen HF. Stigma among health professionals towards patients with substance use disorders and its consequences for healthcare delivery: systematic review. Drug and alcohol dependence. 2013;131(1-2):23-35.
20. Katz IT, Ryu AE, Onuegbu AG, Psaros C, Weiser SD, Bangsberg DR, et al. Impact of HIV-related stigma on treatment adherence: systematic review and meta-synthesis. J Int AIDS Soc. 2013;16(3 Suppl 2):18640.

21. Rueda S, Mitra S, Chen S, Gogolishvili D, Globerman J, Chambers L, et al. Examining the associations between HIV-related stigma and health outcomes in people living with HIV/AIDS: a series of meta-analyses. BMJ Open. 2016;6(7):e011453.
22. Matheson K, Foster MD, Bombay A, McQuaid RJ, Anisman H. Traumatic Experiences, Perceived Discrimination, and Psychological Distress Among Members of Various Socially Marginalized Groups. Front Psychol. 2019;10(416).
23. Wilson EC, Chen Y-H, Arayasirikul S, Raymond HF, McFarland W. The Impact of Discrimination on the Mental Health of Trans*Female Youth and the Protective Effect of Parental Support. AIDS and behavior. 2016;20(10):2203-11.
24. Corrigan PW, Watson AC. Understanding the impact of stigma on people with mental illness. World Psychiatry. 2002;1(1):16-20.
25. Hoyert DL, Miniño AM. Maternal Mortality in the United States: Changes in Coding, Publication, and Data Release, 2018. National vital statistics reports : from the Centers for Disease Control and Prevention, National Center for Health Statistics, National Vital Statistics System. 2020;69(2):1-18.
26. Stern AM. STERILIZED in the Name of Public Health. American Journal of Public Health. 2005;95(7):1128-38.
27. Chambers BD, Arabia SE, Arega HA, Altman MR, Berkowitz R, Feuer SK, et al. Exposures to structural racism and racial discrimination among pregnant and early post-partum Black women living in Oakland, California. Stress and health : journal of the International Society for the Investigation of Stress. 2020;36(2):213-9.
28. Vedam S, Stoll K, Taiwo TK, Rubashkin N, Cheyney M, Strauss N, et al. The Giving Voice to Mothers study: inequity and mistreatment during pregnancy and childbirth in the United States. Reprod Health. 2019;16(1):77.
29. Altman MR, McLemore MR, Oseguera T, Lyndon A, Franck LS. Listening to Women: Recommendations from Women of Color to Improve Experiences in Pregnancy and Birth Care. J Midwifery Womens Health. 2020;65(4):466-73.
30. Afulani PA, Phillips B, Aborigo RA, Moyer CA. Person-centred maternity care in low-income and middle-income countries: analysis of data from Kenya, Ghana, and India. The Lancet Global Health. 2019;7(1):e96-e109.
31. Afulani PA, Sayi TS, Montagu D. Predictors of person-centered maternity care: the role of socioeconomic status, empowerment, and facility type. BMC health services research. 2018;18(1):360.
32. Sudhinaraset M, Treleaven E, Melo J, Singh K, Diamond-Smith N. Women’s status and experiences of mistreatment during childbirth in Uttar Pradesh: a mixed methods study using cultural health capital theory. BMC Pregnancy and Childbirth. 2016;16(1):332.
33. Nyblade L, Stangl A, Weiss E, Ashburn K. Combating HIV stigma in health care settings: what works? J Int AIDS Soc. 2009;12:15.
34. Cook RJ, Dickens BM. Reducing stigma in reproductive health. Int J Gynaecol Obstet. 2014;125(1):89-92.
35. Mohamed D, Diamond-Smith N, Njunguru J. Stigma and agency: exploring young Kenyan women's experiences with abortion stigma and individual agency. Reprod Health Matters. 2018;26(52):1492285.
36. Glenton C, Bohren M, Downe S, Paulsen E, Lewin S, on behalf of Cochrane Effective Practice and Organisation of Care (EPOC). EPOC Qualitative Evidence Synthesis: Protocol and review template. EPOC Resources for review authors. Oslo, Norway: Norwegian Institute of Public Health; 2019.
37. Popay J, Roberts H, Sowden A, Petticrew M, Arai L, Rodgers M, et al. Guidance on the Conduct of Narrative Synthesis in Systematic Reviews. A Product from the ESRC Methods Programme United Kingdom: ESRC; 2006.

38. Braun V & Clarke V. Using thematic analysis in psychology. Qualitative Research in Psychology. 2006;3(2):77-101.
39. Thomas J, Harden A. Methods for the thematic synthesis of qualitative research in systematic reviews. BMC medical research methodology. 2008;8:45.
40. Lewin S, Bohren M, Rashidian A, Munthe-Kaas H, Glenton C, Colvin CJ, et al. Applying GRADE-CERQual to qualitative evidence synthesis findings-paper 2: how to make an overall CERQual assessment of confidence and create a Summary of Qualitative Findings table. Implement Sci. 2018;13(Suppl 1):10.
41. Lewin S, Booth A, Glenton C, Munthe-Kaas H, Rashidian A, Wainwright M, et al. Applying GRADE-CERQual to qualitative evidence synthesis findings: introduction to the series. Implement Sci. 2018;13(Suppl 1):2.
42. Bohren MA, Berger BO, Munthe-Kaas H, Tuncalp O. Perceptions and experiences of labour companionship: a qualitative evidence synthesis. The Cochrane database of systematic reviews. 2019;3:Cd012449.
43. Ames HM, Glenton C, Lewin S. Parents' and informal caregivers' views and experiences of communication about routine childhood vaccination: a synthesis of qualitative evidence. The Cochrane database of systematic reviews. 2017;2:Cd011787.
44. Candy B, King M, Jones L, Oliver S. Using qualitative synthesis to explore heterogeneity of complex interventions. BMC medical research methodology. 2011;11:124.
45. Glenton C, Colvin C, Carlsen B, Swartz A, Lewin S, Noyes J, et al. Barriers and facilitators to the implementation of lay health worker programmes to improve access to maternal and child health: qualitative evidence synthesis. Cochrane Database of Systematic Reviews, in press. 2013.
46. Munthe-Kaas H, Bohren MA, Glenton C, Lewin S, Noyes J, Tunçalp Ö, et al. Applying GRADE-CERQual to qualitative evidence synthesis findings—paper 3: how to assess methodological limitations. Implementation Science. 2018;13(1):9.
47. Colvin CJ, Garside R, Wainwright M, Munthe-Kaas H, Glenton C, Bohren MA, et al. Applying GRADE-CERQual to qualitative evidence synthesis findings—paper 4: how to assess coherence. Implementation Science. 2018;13(1):13.
48. Glenton C, Carlsen B, Lewin S, Munthe-Kaas H, Colvin CJ, Tunçalp Ö, et al. Applying GRADE-CERQual to qualitative evidence synthesis findings—paper 5: how to assess adequacy of data. Implementation Science. 2018;13(1):14.
49. Noyes J, Booth A, Lewin S, Carlsen B, Glenton C, Colvin CJ, et al. Applying GRADE-CERQual to qualitative evidence synthesis findings–paper 6: how to assess relevance of the data. Implementation Science. 2018;13(1):4.

Appendix 1

Search strategies to be added
