# Supplementary material for: Strategies to reduce stigma and discrimination in sexual and reproductive healthcare settings: A mixed-methods systematic review
Source: PLOS Glob Public Health. 2022 Jun 15;2(6):e0000582. doi: 10.1371/journal.pgph.0000582 (PMC10021469; doi:10.1371/journal.pgph.0000582)
Supplement: S2 Text — (PDF) [file pgph.0000582.s005.pdf]

S3 Appendix. Search strategies

**Medline (6,870 results - April 6, 2022)**	
**1**	Reproductive Health/ or Sexual Health/ or Pregnancy/ or Reproductive Behavior/ or Gender-Based Violence/ or Sexually Transmitted Diseases/ or Sexual Behavior/ or Delivery, Obstetric/ or Parturition/ or Maternal Health/ or Abortion,Induced/ or Contraception/ or Family Planning Services/
**2**	("sexual and reproductive health*" or pregnan* or childbirth or birth or reproductive behavio* or sexual health* or reproductive health* or reproductive choice or abortion or reproductive coercion or infertility or sexually transmitted infection* or sexually transmitted disease* or gender-based violence or reproductive cancer* or termination or TOP or contraception or family planning or chlamydia or gonorrhea or gonorrhea or trichomoniasis or genital warts or genital herpes or herpes simplex virus or pubic lice or scabies or syphilis or bacterial vaginosis or human papillomavirus or HPV).ti,ab.
**3**	1 or 2
**4**	Social Discrimination/ or Social Stigma/ or Stereotyping/ or Racism/ or Ageism/ or Homophobia/ or Sexism/ or Xenophobia/ or Prejudice/
**5**	(discriminat* or stigma* or prejudice or mistreat* or injustice or stereotyping or victim* or racism or ageism or agism or homophobi* or bias or xenophobi* or ableism or ablism of transphobi* or sexism or misogyn* or inequit* or inequalit* or disparit* or prejudic* or oppress* or cultural* safe* or cultural* aware* or cultural* competen* or cultural* responsive* or cultural* sensitiv* or cultural* target* or cultural* tailor* or cultural* appropriat* or cultural* humilit* or cultural* securit* or self-determination or strength* based).ti,ab.
**6**	4 or 5
**7**	exp Reproductive Health Services/ or exp Women's Health/ or Reproductive Health/
**8**	(((health or healthcare or health care) adj3 (system* or facilit* or service* or setting*)) or (clinic or clinics)).ti,ab.
**9**	7 or 8
**10**	(intervention* or program* or workshop* or participatory or education or training or policies or policy or strategy or strategies or initiative or model* of care or follow-up care or project or programme* or protocol* or action plan or law or inclusiv*).ti,ab.
**11**	3 and 6 and 9 and 10

CINAHL (2,431 results – March 29, 2022)	
**S1**	(((MM "Reproductive Health" or MM "Sexual Health" or MM "Pregnancy" or MM "Gender-Based Violence" or MM "Sexually Transmitted Diseases" or MM “Delivery, Obstetric” or MM “Labor” or MM “Abortion, Induced” or MM Contraception or MM “Contraception” or MM “Family Planning”)) OR (TI ("sexual and reproductive health*" or pregnan* or childbirth or "reproductive behavio*" or "sexual health*" or "reproductive health*" or "reproductive choice" or abortion or "reproductive coercion" or infertility or "sexually transmitted infection*" or "sexually transmitted disease*" or "gender-based violence" or "reproductive cancer*" or termination or TOP or contraception or family planning or chlamydia or gonorrhoea or gonorrhea or trichomoniasis or “genital warts” or “genital herpes” or “herpes simplex virus” or “pubic lice” or scabies or syphilis or “bacterial vaginosis” or “human papillomavirus” or HPV) OR AB ("sexual and reproductive health*" or pregnan* or childbirth or "reproductive behavio*" or "sexual health*" or "reproductive health*" or "reproductive choice" or abortion or "reproductive coercion" or infertility or "sexually transmitted infection*" or "sexually transmitted disease*" or "gender-based violence" or "reproductive cancer*" or termination or TOP or contraception or “family planning” or chlamydia or gonorrhoea or gonorrhea or trichomoniasis or “genital warts” or “genital herpes” or “herpes simplex virus” or “pubic lice” or scabies or syphilis or “bacterial vaginosis” or “human papillomavirus” or HPV)))
**S2**	(((MM "Discrimination" of MM "Stigma" or MM "Stereotyping" or MM “Racism” or MM “Ageism” or MM “Homophobia” or MM “Sexism” or MM “Prejudice”)) OR (TI (discriminat* or stigma* or prejudice or mistreat* or injustice or stereotyping or victim* or racism or ageism or agism or homophobi* or bias or xenophobi* or ableism or ablism of transphobi* or sexism or misogyn* or inequit* or inequalit* or disparit* or prejudic* or oppress* or “cultural* safe*” or “cultural* aware*” or “cultural* competen*” or “cultural* responsive*” or “cultural* sensitiv*” or “cultural* target*” or “cultural* tailor*” or “cultural* appropriat*” or “cultural* humilit*” or “cultural* securit*” or self-determination or “strength* based”) or AB (discriminat* or stigma* or prejudice or mistreat* or injustice or stereotyping or victim* or racism or ageism or agism or homophobi* or bias or xenophobi* or ableism or ablism of transphobi* or sexism or misogyn* or inequit* or inequalit* or disparit* or prejudic* or oppress* or “cultural* safe*” or “cultural* aware*” or “cultural* competen*” or “cultural* responsive*” or “cultural* sensitiv*” or “cultural* target*” or “cultural* tailor*” or “cultural* appropriat*” or “cultural* humilit*” or “cultural* securit*” or self-determination or “strength* based”)))
**S3**	(((TI ((health or healthcare or "health care") N3 (system* or facilit* or service* or setting*))) OR (AB ((health or healthcare or "health care") N3 (system* or facilit* or service* or setting*)))) OR (TI (clinic or clinics) or AB (clinic or clinics)))
**S4**	(TI (intervention* or program* or workshop* or participatory or education or training or policies or policy or strategy or strategies or initiative or model* of care or follow-up care or project or programme* or protocol* or “action plan” or law or inclusiv*) OR AB (intervention* or program* or workshop* or participatory or education or training or policies or policy or strategy or strategies or initiative or model* of care or follow-up care or project or programme* or protocol* or “action plan” or law or inclusiv*))
**S5**	S1 AND S2 AND S3 AND S4

Global Health Ovid (2,600 results – April 13, 2022)	
**1**	(reproductive health or reproductive behaviour or sexual health or pregnancy or sexually transmitted diseases or sexually transmitted infections or sexual behaviour or parturition or abortion or contraception or family planning).hw.
**2**	("sexual and reproductive health*" or pregnan* or childbirth or "reproductive behavio*" or "sexual health*" or "reproductive health*" or "reproductive choice" or abortion or "reproductive coercion" or infertility or "sexually transmitted infection*" or "sexually transmitted disease*" or "gender-based violence" or "reproductive cancer*" or termination or TOP or contraception or "family planning" or chlamydia or gonorrhoea or gonorrhea or trichomoniasis or "genital warts" or "genital herpes" or "herpes simplex virus" or "pubic lice" or scabies or syphilis or "bacterial vaginosis" or "human papillomavirus" or HPV).ti,ab.
**3**	1 or 2
**4**	(social stigma or discrimination).hw.
**5**	(discriminat* or stigma* or prejudice or mistreat* or injustice or stereotyping or "victim* racism" or ageism or agism or homophobi* or bias or xenophobi* or ableism or ablism or transphobi* or sexism or misogyn* or inequit* or inequalit* or disparit* or prejudic* or oppress* or "cultural* safe*" or "cultural* aware*" or "cultural* competen*" or "cultural* responsive*" or "cultural* sensitiv*" or "cultural* target*" or "cultural* tailor*" or "cultural* appropriat*" or "cultural* humilit*" or "cultural* securit*" or "self-determination" or "strength* based").ti,ab.
**6**	4 or 5
**7**	((health or healthcare or "health care") adj3 (system* or facilit* or service* or setting*)).ti,ab.
**8**	(clinic or clinics).ti,ab.
**9**	7 or 8
**10**	(intervention* or program* or workshop* or participatory or education or training or policies or policy or strategy or strategies or initiative or "model* of care" or "follow-up care" or project or programme* or protocol* or "action plan" or law or inclusiv*).ti,ab.
**11**	3 and 6 and 9 and 10
