# Supplementary material for: Strategies to reduce stigma and discrimination in sexual and reproductive healthcare settings: A mixed-methods systematic review
Source: PLOS Glob Public Health. 2022 Jun 15;2(6):e0000582. doi: 10.1371/journal.pgph.0000582 (PMC10021469; doi:10.1371/journal.pgph.0000582)
Supplement: Attachment — Submitted filename: Response to reviewers_03-30-2022mb.docx [file pgph.0000582.s006.docx]

Dear Editors and Reviewers,

Many thanks for the constructive feedback on our paper. We have addressed the point-by-point feedback below and with revisions in a tracked changes version of the manuscript. We appreciate this feedback and believe that it has strengthened our paper.

We also note that we have conducted an update of the 3 database searches given that the previous searches were run in October/November 2020. All searches are now up-to-date to March/April 2022.

Sincerely,

Meghan Bohren, on behalf of the authors

1. Does this manuscript meet PLOS Global Public Health’s publication criteria? Is the manuscript technically sound, and do the data support the conclusions? The manuscript must describe methodologically and ethically rigorous research with conclusions that are appropriately drawn based on the data presented.

Reviewer #1: Yes

Reviewer #2: Yes

2. Has the statistical analysis been performed appropriately and rigorously?

Reviewer #1: N/A

Reviewer #2: N/A

3. Have the authors made all data underlying the findings in their manuscript fully available (please refer to the Data Availability Statement at the start of the manuscript PDF file)? The PLOS Data policy requires authors to make all data underlying the findings described in their manuscript fully available without restriction, with rare exception. The data should be provided as part of the manuscript or its supporting information, or deposited to a public repository. For example, in addition to summary statistics, the data points behind means, medians and variance measures should be available. If there are restrictions on publicly sharing data—e.g. participant privacy or use of data from a third party—those must be specified.

Reviewer #1: Yes

Reviewer #2: Yes

4. Is the manuscript presented in an intelligible fashion and written in standard English?

PLOS Global Public Health does not copyedit accepted manuscripts, so the language in submitted articles must be clear, correct, and unambiguous. Any typographical or grammatical errors should be corrected at revision, so please note any specific errors here.

Reviewer #1: Yes

Reviewer #2: Yes

| Reviewer feedback | Authors’ response |
| --- | --- |
| REVIEWER 1 |  |
| General comment: Thank you for the opportunity to read and review this paper. The authors are to be commended for this systematic review of interventions designed to reduce stigma and discrimination in sexual and reproductive healthcare settings. This paper is of international public health relevance, all people have the right to access SRH services that are free from stigma and discrimination which have significant negative impacts on the SRH and wellbeing of individuals in all parts of the world.  The authors set out to review the evidence for strategies and interventions that aim to reduce stigma and or discrimination in sexual and reproductive health care settings, taking into account the multiple levels on which stigma operates. They used a narrative synthesis approach which is appropriate for the topic area with both quantitative and qualitative studies included in the review. No meta-analyses or other statistical analyses were undertaken due to the heterogeneity of outcome measurement in studies reviewed. Having identified 7000+ papers, only 12 met inclusion criteria (9 included quantitative aspects and 3 qualitative) - which is a clear indicator that more work is urgently needed in this area.  The authors have clearly summarized the findings of included studies, and also highlight the gaps and opportunities for future research. They have suggested the ways in which future researchers could consider different types of stigma and highlight the importance of involving the communities of interest in the design of such work. Another important message is that designing interventions to improve outcomes for marginalized groups without specific consideration of stigma and discrimination or measuring these, is unlikely to succeed. | Thank you for this feedback. |
| INTRODUCTION  Comment: This provides an informative and well-written description of the relevant terms, impacts and understandings of stigma and discrimination as they relate to SRH services and outcomes. Referencing is thorough and current. | Thank you. |
| METHODS  Comment: The methods are well described and in enough detail for others to follow or replicate. Information recommended for inclusion in the PRISMA checklist has been included in sufficient detail. The risk of bias has been acknowledged and discussed in the paper and further details included in S4 appendix. The study protocol has been provided and appears to align with the work undertaken.  Minor queries:  1. P8, lines 188-190. ‘We excluded STI and HIV counseling, testing, and treatment services if conducted outside the context of contraceptive or maternal health services, as these topics have been well documented in the literature (15, 27, 28). Can the authors please clarify this sentence reads as intended – many STI and HIV testing and treatment services would be separate to contraceptive or maternal health services and I’m not sure that we already know how to address/reduce stigma and discrimination for these aspects of SRH. Refs 27 and 28 are about HIV-related stigma, whereas Nyblade et al are looking at stigma across a wide range of health conditions including HIV but not SRH specifically.  2. It isn’t immediately clear why S5 appendix includes only two of the qualitative studies – does the table just include studies where concerns about biases arose (whereas the other studies were assessed and deemed free from biases?) Please clarify this for readers. | Thank you.  The systematic reviews referenced here cover the HIV and STI literature on stigma in HIV/STI healthcare setting extensively. We therefore decided to exclude these topics from our review as we believed it would be duplicative of existing work.  The 3 studies included in S5 appendix of critical appraisal of qualitative studies are the 3 included studies that used only qualitative methods. The Harris 2011 and Debbink 2016 papers are reported and assessed together, as they are from the same study. The remaining studies report on quantitative measures and the critical appraisals are reported in S4 Appendix. No revisions made. |
| RESULTS  Comment: Very good use of tables and figures to summarize and collate the review findings, all aspects in the PRISMA checklist relating to results are covered. Figure 1 is excellent and Figure 4 is a really effective way to show what type(s) of stigma the different studies attempt to address.  Minor suggestions:  3. Tables 1 and 2 – It would be helpful to have the reference numbers included alongside author names to make it easier for readers to match up the written summary of studies (on p11, and p 16) with the details in the tables  4. Figure 3 – Please add in reference numbers alongside Author/year. | Thank you.  We have added references to Tables 1 and 2, and Figure 3. |
| DISCUSSION  Key findings are well summarized and implications for future research discussed. Many studies have described stigma and discrimination related to SRH, but few studies have attempted to address this challenging topic. This paper will help other researchers to conceptualize the way(s) in which they might go about setting up an intervention to tackle one or more levels of SRH-related stigma and discrimination. | Thank you. |
| REVIEWER 2 |  |
| Thank you for the opportunity to review this manuscript which reports on a mixed-methods systematic review of strategies to reduce stigma and discrimination in healthcare settings offering sexual and reproductive care. My comments include suggestions for some textual modifications in the manuscript and methodological and analytical queries regarding the data and findings presented.  Abstract  Reads very well. | Thank you |
| Introduction  Page 3: Lines 8-9  Suggest re-phrasing as “The 2030 Sustainable Development Agenda highlights two targets specific to achieving universal access to sexual and reproductive health, health-care services and reproductive rights”. As presently phrased, it might be understood that the SDG targets also encompasses “sexual rights”, which isn’t the case. | We have made this revision to clarify the SDG agenda. See page 3 lines 8-9. |
| The introduction and brief discussion of “Reproductive Justice” as a discourse that identifies the intersecting structural factors contributing to inequities in SRHR outcomes can be better contextualised for the reader and its relevance to the topic of this manuscript more clearly spelt out. For example, the international community is likely very familiar with the discourse on human rights, hence providing some background on why or how an RJ approach extends or builds upon or addresses the limitations of a human rights framework to achieving SRHR would be beneficial information to include. I do think the discussion on RJ would be better placed once an overview of the various types of stigma are presented (Figure 1). RJ can be a useful lens through which to understand how structural stigma can emerge through the intersecting influences of law and social policy that have contributed to longstanding structural oppression and injustice. Doing so, can guide the reader to look beyond the inter-personal aspects of stigma and implicating the institutions and systems that perpetuate it at a structural level. | Thank you for this very helpful comment. We have moved the discussion about RJ to after the introduction of figure 1 (page 5, lines 71-84 in tracked change version):  Reproductive justice, a movement led by women of color in the United States, strives to achieve health equity, end discrimination and oppression, and challenge medical hierarchies through the recognition that the intersectionality of social, political and economic identities shape peoples’ abilities to access safe, appropriate, and respectful SRHR services (3, 4). Through the lens of reproductive justice, we can better understand the intersecting influences of laws and social policies on people’s and communities’ health and well-being, and how these laws and policies contribute to longstanding injustices and structural oppression. Tantamount to achieving reproductive justice is acknowledging, then dismantling oppressive systems that prohibit all people from achieving their fullest potential. Reproductive justice, therefore, can extend conceptualizations of stigma and discrimination from the interpersonal level, to better implicate the institutions and systems that perpetuate stigma and discrimination at the structural level. Similarly, global evidence has shown that women are mistreated during childbirth, which includes both structural and interpersonal discrimination while seeking care (5). Eliminating stigma and discrimination within healthcare settings is critical to the attainment of reproductive justice and respectful care for all. |
| Stigma and discrimination in healthcare settings  Page 4: Lines 51-52  The phrase “socially undesirable” in the explanation for Enacted stigma is confusing. Instead suggest re-phrasing as “Enacted stigma refers to the manifestations of unfair treatment arising from adverse social judgment” | Thank you for the suggestion, we have made this change (tracked changes page 4, line 52-53). |
| Figure 1 Page 34  This is a useful visual to depict the various types of stigma and how they manifest in individuals, the health system and society. A few related queries and suggestions:  Within “Perceived Stigma”, it is unclear to me how the following three components are conceptually distinct to necessitate separate labels:  1. “Healthcare provider perspectives of individual or group attributes (e.g., gender identity, homophobia, racism)”  2. “Healthcare provider perspectives and internalized belief systems of individuals or groups”  3. “Internalised belief systems”  Instead, I would suggest combining the above into the following: “Healthcare provider perspectives and belief systems regarding individual or group attributes “(e.g., related to gender identity, sexual orientation, racial group etc.,).  I would also recommend having “Internalised belief systems” as a stand-alone component of “Internalised Stigma”, as you indicate in the text that this form of stigma is applied by the individual onto themselves, which is likely related to their own belief systems, shaped by societal norms and expectation.  “Labelling, stereotyping, othering” which are the processes by which stigma is manifested seems to fit better in “Enacted Stigma” than “Perceived Stigma”  Within “Enacted Stigma” rather than having “poor person-centered care” as a component with examples, I would include the examples themselves as separate components within this domain as they are sufficiently descriptive to indicate that these actions constitute poor person-centered care. If the authors decide to keep “poor person-centered care” I suggest at least having “Denial of care” as a separate component since this is conceptually different from the provision of sub-standard care, as the latter assumes that some type of care is provided.  In Structural Stigma, “Restrictive access to health services (e.g., abortion, contraception, fertility)” is repeated twice. | Thank you, we have made these revisions. |
| Strategies to reduce stigma and discrimination in healthcare settings  Line 127: Add colon after follows: | We have made this change (tracked changes version page 7, line 142). |
| METHODS  Types of studies, Page 7, lines 158-161.  In describing their operational definition of “strategy”, the authors combine aspects of the study design (e.g., mystery client methodology, non-randomised designs) with the strategies themselves (e.g., workshops, training, simulation etc). This is confusing because I view the two components as distinct. The earlier sentence in this paragraph (156-158) explains the type of study designs that were considered eligible for inclusion. In the operational definition of strategy, I would recommend that the authors focus on what type of strategies were considered eligible, i.e., policy reform, healthcare provider training, community-based approaches targeting healthcare users etc. | Thank you, we have clarified this (tracked changes version page 8, line 174-177):  We developed an operational definition of what constitutes a ‘strategy’ that was inclusive of strategies such as policy reform, introducing new models of care, healthcare provider training (workshops, sensitization training, simulation), mystery clients, and community-based approaches targeting healthcare users. |
| Topics of interest, types of participants, and settings Page 8, Lines 178-187.  I commend the authors for using the Guttmacher-Lancet Commission’s definition of SRHR. Included in the Commission’s definition of the package of SRHR services but missing from the manuscript are (a) prevention, identification, and counselling for gender-based violence (outside of maternal health programs), (b) comprehensive sexuality education, and (c) treatment of infertility. Can the authors comment on why this is the case, and include this in the text? | Thank you for this, we have realized that our description in the manuscript was insufficient and confusing to the reader. We included search terms for both gender-based violence and infertility (see search strategies). However we note that we did not identify any studies relevant for inclusion. We also included a search term for ‘sexual health’; however, we did not include search terms for ‘comprehensive sexuality education,’ nor were studies about comprehensive sexuality education eligible for inclusion, as it is typically offered in school-based settings, rather than healthcare settings (the eligibility criteria for ‘settings’). We have revised this in the tracked changes version, page 9, rows 199-211. |
| Data management, analysis and synthesis  Lines 241-245  In describing the approach to narrative synthesis, the authors discuss evaluating how “critical design factors” and “relationships between study designs” influenced the outcomes of interest. Can the authors clarify here and, in the text, whether this refers to the design of the study itself (e.g., quasi-experimental designs, RCTs, qualitative study etc.,) or the design of the intervention or both? | We considered both the study design and intervention design factors in this stage, and clarified this in the text (tracked changes page 10 line 258):  Second, we explored the relationships between and across studies to understand how critical study design and intervention design factors may have influenced the likelihood of implementation success. |
| Description of interventions  Page 16, lines 28-30  The authors note that one-half of the interventions included repeated components whereas the remaining were once-off sessions; were there any noticeable differences in the impact based on differential “doses” of the intervention? The authors may want to comment on any differences or lack thereof in the text. | Thank you for raising this interesting point. We considered how we could assess the differences in intervention effects based on the “doses” of the intervention (once-off, or repeat). However, we found that due to the heterogeneity in outcomes reported, and multiple (and often many) outcomes reported in the quantitative studies, we are unable to conclude anything meaningful about the impact of a once-off versus repeat training on outcomes of interest. We hypothesize that given the complexity of addressing stigma and discrimination, a once-off training would be less likely to evoke change (especially sustainable change), and that measurement of this in the included studies is limited by evaluation of intervention impact typically occurring close to the time of the intervention. This may be particularly true for interventions targeting areas other than increasing provider awareness, which we hypothesize would be likely to need repeat and sustained engagement with stakeholders to influence systems change. We have added this to the discussion section “limitations and strengths”. |
| Quantitative evaluation of impact  Page 19, lines 50-51  The authors mention that there are “limitations” in whether information-based strategies for stigma reduction translated into changed practice or increased confidence in care. Could these limitations be described in brief?  I commend the authors in conducting a risk of bias assessment for the quantitative studies but would also like the authors to comment in this section whether any aspects of the “serious” and “critical” assessments of bias for all included studies are important to note in the context of how “valid” the reported results are likely to be, especially for those studies where an “effect” of the intervention is reported. | We have addressed this in two places. First, in tracked changes version page 21 line 55-56 we have added: “…as these outcomes were typically not measured or reported by either providers or users.”  We have also added to tracked change version page 21 lines 65-68: “Of note, the three articles that evaluated user experiences after the intervention collected data ranging from approximately two to six months after intervention (63), six months after the intervention (62), and six and twelve months after the intervention (59), which limits the evaluation of the sustainability of the intervention effects over time.”  We have expanded the section reporting on risk of bias on tracked change version page 17, lines 8-13: Most (8/9) studies had an overall critical risk of bias, primarily due to bias from confounding (typically due to no control group or adjustment for potential confounders), selection of participants (typically due to self-selection into the intervention group), bias due to missing data (substantial loss to follow-up or blank surveys) and measurement of outcomes (typically because participants who knew they were in the intervention group reported the impact of the intervention on their knowledge or behavior). |
| DISCUSSION  Thorough and well-written. | Thank you. |
